# Quantitative measurement of pathogen specific human memory T cell repertoire diversity using a CDR3β-specific microarray

**DOI:** 10.1186/1471-2164-8-329

**Published:** 2007-09-19

**Authors:** Xujing Wang, Shuang Jia, Lisa Meyer, Maryam B Yassai, Yuri N Naumov, Jack Gorski, Martin J Hessner

**Affiliations:** 1 The Max McGee National Research Center for Juvenile Diabetes, Department of Pediatrics, The Medical College of Wisconsin and The Children's Hospital Research Institute of Children's Hospital of Wisconsin, 8701 Watertown Plank Road, Milwaukee, WI 53226, USA; 2 The Human and Molecular Genetics Center, The Medical College of Wisconsin, 8701 Watertown Plank Road, Milwaukee, WI 53226, USA; 3 Department of Pathology, University of Massachusetts Medical School, 55 Lake Avenue North, Worcester, MA 01655, USA; 4 The Blood Research Institute, BloodCenter of Wisconsin, 8727 Watertown Plank Road, Milwaukee, Wisconsin 53226, USA

## Abstract

**Background:**

Providing quantitative microarray data that is sensitive to very small differences in target sequence would be a useful tool in any number of venues where a sample can consist of a multiple related sequences present in various abundances. Examples of such applications would include measurement of pseudo species in viral infections and the measurement of species of antibodies or T cell receptors that constitute immune repertoires. Difficulties that must be overcome in such a method would be to account for cross-hybridization and for differences in hybridization efficiencies between the arrayed probes and their corresponding targets. We have used the memory T cell repertoire to an influenza-derived peptide as a test case for developing such a method.

**Results:**

The arrayed probes were corresponded to a 17 nucleotide TCR-specific region that distinguished sequences differing by as little as a single nucleotide. Hybridization efficiency between highly related Cy5-labeled subject sequences was normalized by including an equimolar mixture of Cy3-labeled synthetic targets representing all 108 arrayed probes. The same synthetic targets were used to measure the degree of cross hybridization between probes. Reconstitution studies found the system sensitive to input ratios as low as 0.5% and accurate in measuring known input percentages (R^2 ^= 0.81, R = 0.90, p < 0.0001). A data handling protocol was developed to incorporate the differences in hybridization efficiency. To validate the array in T cell repertoire analysis, it was used to analyze human recall responses to influenza in three human subjects and compared to traditional cloning and sequencing. When evaluating the rank order of clonotype abundance determined by each method, the approaches were not found significantly different (Wilcoxon rank-sum test, p > 0.05).

**Conclusion:**

This novel strategy appears to be robust and can be adapted to any situation where complex mixtures of highly similar sequences need to be quantitatively resolved.

## Background

Over the past decade, microarrays have evolved beyond high-throughput gene expression profiling to a wide variety of applications that include genotyping and resequencing. Even more diverse are the biological venues where these new developments have been applied. This report describes a novel array-based assay that accurately identifies and quantifies nucleotide sequences that share extensive identity. Such an approach has numerous applications including human leukocyte antigen (HLA) system, viral or bacterial genotyping, characterization microbial species within environmental samples, or as described here, the measurement of pathogen specific human memory T-cell repertoire diversity.

During the initiation of immune responses, individual T-cell clones recognize peptides presented by major histocompatibility complex (MHC) molecules through T-cell receptors (TCR). TCRs are heterodimers consisting of either α and β chains or γ and δ chains. Each chain is composed of variable (V), diversity (D, β and δ chains), joining (J), and constant (C) regions encoded by gene segments that undergo rearrangement during thymic T-cell development [1]. The shape and charge, and therefore TCR specificity is determined by the selection of the V, D and J genes, as well as the rearrangement process itself where nucleotides at junctions of the V, D, and J segments are added or removed. The portion of the TCR encoded at the rearrangement site is referred to as the third complementarity-determining region (CDR3). Any rearrangement utilizing the same V and J genes is identical except for the unique nucleotide sequence of the CDR3. This sequence is considered to define a clonotype and serves as a fingerprint for the T-cell lineage bearing it. The frequency with which a particular TCR clonotype is encountered can be taken to be a measure of clonal expansion.

The amino acid sequence encoded by CDR3 conveys fine antigen specificity. Often recognition of a particular antigen-MHC complex is mediated by TCR using identical V chains with very similar CDR amino acid sequences (reviewed in [2]). Because of the degeneracy of the genetic code this can lead to responses characterized by multiple clonotypes that encode identical CDR3 amino acid sequences.

Since antigen specific T-cell responses are central to human immunity, and there is significant interest in the relationship between lymphocyte diversity and immunocompetence, the analysis of T-cell repertoires is highly relevant [3,4]. An individual α/β repertoire consists of an estimated 10^6 ^β chains, each pairing with a limited number of α chains, thus the complexity of the TCR makes analysis cumbersome [1,5,6]. Molecular genetic approaches have been developed for analysis of TCR repertoires that involve amplification of cDNA using C and V specific primers, or genomic DNA between V and J specific primers, followed by separation of PCR products through denaturing gel electrophoresis. This approach, termed spectratyping, provides an estimation of the abundance of a particular V gene family relative to others as well as the different TCR based on their CDR3 length [7-10]. The highest resolution method for measuring and identifying T-cells within repertoires is to generate sequences of the CDR3. This is known as clonotyping and can be accomplished by using PCR to amplify variable families from cDNA followed by sub-cloning and sequencing the product to study repertoire structure [8]. Such approaches have been found to give an unbiased measurement of TCR diversity [11].

We have been studying the cytotoxic memory T-cell response to influenza virus [8,12-14]. The M1-derived epitope, M_158–66_, an immunodominant peptide derived from influenza A virus, induces a strong CD8 T-cell response in HLA-A2 individuals [3,4]. M_158–66 _bound to HLAA2.1 (M1-A2) is preferentially recognized by CD8 T-cells expressing β variable 17 chains (VB17) rearranged to JB2.7 chains and possessing CDR3 regions with an "IRSS" amino acid motif. We have also observed that the memory repertoires are complex consisting of a few clonotypes observed often, many clonotypes observed once and others present in intermediate abundances. Such complexity in memory repertoires implies a selection for multiple T-cell lineages and belies a simple selection process. We have already shown that different clonotypes may be needed at different stages in an immune response relative to pathogen density [12]. Further work is required to study how this repertoire complexity changes with time, exposure to pathogen or immunization in a population of HLA-A2 individuals.

In cases such as this, where a complex repertoire exhibits an identified, highly prevalent CDR3 amino acid motif, with individual clonotypes observed at different frequencies, an alternate high resolution approach to sub cloning and sequencing of family specific TCR VB PCR products would be to generate microarrays that represent all possible encoding of a particular motif. Such an approach would enable a rapid, high-throughput measurement of clontypic diversity at the nucleotide level, thereby facilitating population-based studies aimed at better understanding the processes of memory repertoire selection. Described here is a microarray assay that accurately identifies and quantifies T-cell repertoires for VB17 – JB2.7 rearrangements possessing IRSS as the core amino acid sequence. Because the final serine in the motif is often encoded by the J region gene, the actual number of possibilities encoding most encountered IRSS motifs is 108 (3 × 6 × 6).

The described assay represents the foundation of an expanding system to quantitatively measure a broader range of the memory repertoire that greatly simplifies the process of sampling immune repertoires in large populations over time. This specific application is directly relevant to laboratories investigating T-cell receptor usage, however, the overall strategy is applicable for quantitative genotyping of any complex mixture or highly polymorphic system where the constituent sequences differ by only one or a few nucleotides.

## Results

Previously, we observed that T-cells of HLA-A2 individuals responding to influenza M1 peptide utilized TCR rearrangements between the JB2.7 and VB17 gene families possessing CDR3 regions encoding an "IRSS" amino acid motif [12]. Therefore, our goal was to develop an oligonucleotide based-microarray for typing the presence and abundance of 108 CDR3β sequences encoding IRSS as the core amino acid motif. These 108 clonotypic sequences from VB17 – JB2.7 expressing T cells account for 21.7% of the memory repertoire in 40 individuals analyzed thus far.

**Figure 1:**
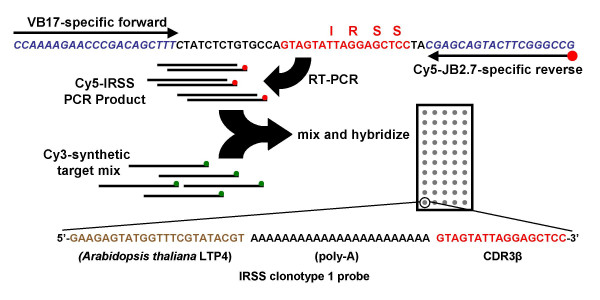
Schematic of overall assay design. Briefly human cDNA is amplified in a non saturating PCR reaction possessing VB17 and Cy-5 labeled JB2.7-specific primers. Purified PCR products are combined with an equimolar mix of synthetic targets complementary to all 108 IRSS-specific CDR3β probes on the array and hybridized, washed and analyzed. Probe design is illustrated at the bottom of the figure.

**Figure 2:**
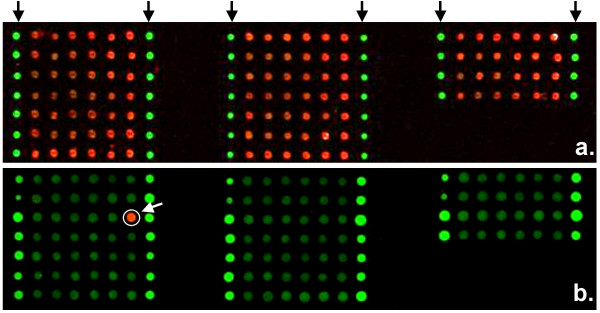
Hybridization of CDR3β-specific microarrays. Panel A. Cy3/FITC overlay image of Cy3-labeled *A. thaliana *LTP4 synthetic target (red) hybridized to 108 CDR3β-specific probes. Black arrows indicate negative controls, printing buffer with FITC-labeled tracking oligonucleotide (green) but no CDR3β-specific probe. Panel B. Cy3/FITC overlay image of Cy3-labeled IRSS clonotype 21 synthetic target (red, indicated by white arrow) hybridized to 108 CDR3β-specific probes. Outer columns of each subarray are negative controls. Note hybridization specificity.

The assay design is illustrated in Figure 1. The array is comprised of 108 63-mer probes. The probes were designed with a 23 nucleotide 5'-region specific for the *Arabidopsis thaliana *LTP4 gene; a 23 nucleotide polyA spacer, and a 17 nucleotide IRSS-specific region (sense strand) placed at the 3'-end of the probe. The *A. thaliana *specific region offers advantages in terms of quality control. First, it makes possible the hybridization of a single labeled complementary *A. thaliana *synthetic target to all elements of the array (Figure 2A). Second, since oligonucleotides are synthesized in a 3'→5' direction, robust *A. thaliana *signal provides control to the synthesis quality of the more important 3' CDR3β-specific region. This approach, combined with the use of a labeled tracking oligonucleotide that is co-spotted with the analyte-specific 63-mers [15], provides control of both probe synthesis and array fabrication.

### Specificity of 108 IRSS-specific CDR3β probes

Specificity is a major issue in any genotyping assay. Previously, we have found short oligonucleotides useful in resolving alleles sharing extensive identity [16,17]. Thus, we empirically optimized the length of the CDR3β-specific probe region to 17 nucleotides (data not shown). In order to test the specificity of each of the 108 probes, complementary synthetic targets (73-mers) were designed to precisely mimic the anti-sense strand of RT-PCR products generated by the VB17 and JB2.7-specific oligonucleotide primers used for clonotyping.

The 108 IRSS clonotype sequences possess high degrees of identity, with any single clonotype differing by only one or a few nucleotides from any other (Table 1). Thus, the degree of cross-hybridization and its potential impact on identifying and quantifying specific CDR3β sequences was investigated. In general the measured intensity (*M*_*i *_*) *of a probe *i *can be written as:

**Table 1:** Partial probe and synthetic target sequences

**IRSS**	Probe*	Synthetic Target**	**IRSS**	Probe*	Synthetic Target**
1	-GTAGTATTAGGAGCTCC-3'	-GGAGCTCCTAAT-	55	-GTAGTATCCGCTCCTCC-3'	-GGAGGAGCGGAT-
2	-GTAGTATAAGGAGCTCC-3'	-GGAGCTCCTTAT-	56	-GTAGTATTCGATCCTCC-3'	-GGAGGATCGAAT-
3	-GTAGTATAAGAAGCTCC-3'	-GGAGCTTCTTAT-	57	-GTAGTATCCGATCCTCC-3'	-GGAGGATCGGAT-
4	-GTAGTATCAGGAGCTCC-3'	-GGAGCTCCTGAT-	58	-GTAGTATTCGGTCCTCC-3'	-GGAGGACCGAAT-
5	-GTAGTATTAGAAGCTCC-3'	-GGAGCTTCTAAT-	59	-GTAGTATCCGGTCCTCC-3'	-GGAGGACCGGAT-
6	-GTAGTATTCGTAGCTCC-3'	-GGAGCTACGAAT-	60	-GTAGTATTAGGAGTTCC-3'	-GGAACTCCTAAT-
7	-GTAGTATCCGCAGCTCC-3'	-GGAGCTGCGGAT-	61	-GTAGTATCAGGAGTTCC-3'	-GGAACTCCTGAT-
8	-GTAGTATAAGGTCCTCC-3'	-GGAGGACCTTAT-	62	-GTAGTATTAGAAGTTCC-3'	-GGAACTTCTAAT-
9	-GTAGTATAAGATCCTCC-3'	-GGAGGATCTTAT-	63	-GTAGTATCAGAAGTTCC-3'	-GGAACTTCTGAT-
10	-GTAGTATACGTAGCTCC-3'	-GGAGCTACGTAT-	64	-GTAGTATTAGGTCTTCC-3'	-GGAAGACCTAAT-
11	-GTAGTATACGCAGCTCC-3'	-GGAGCTGCGTAT-	65	-GTAGTATCAGGTCTTCC-3'	-GGAAGACCTGAT-
12	-GTAGTATACGAAGCTCC-3'	-GGAGCTTCGTAT-	66	-GTAGTATTAGATCTTCC-3'	-GGAAGATCTAAT-
13	-GTAGTATACGGAGCTCC-3'	-GGAGCTCCGTAT-	67	-GTAGTATCAGATCTTCC-3'	-GGAAGATCTGAT-
14	-GTAGTATACGTTCCTCC-3'	-GGAGGAACGTAT-	68	-GTAGTATTCGTAGTTCC-3'	-GGAACTACGAAT-
15	-GTAGTATACGCTCCTCC-3'	-GGAGGAGCGTAT-	69	-GTAGTATCCGTAGTTCC-3'	-GGAACTACGGAT-
16	-GTAGTATACGATCCTCC-3'	-GGAGGATCGTAT-	70	-GTAGTATTCGCAGTTCC-3'	-GGAACTGCGAAT-
17	-GTAGTATACGGTCCTCC-3'	-GGAGGACCGTAT-	71	-GTAGTATCCGCAGTTCC-3'	-GGAACTGCGGAT-
18	-GTAGTATAAGGAGTTCC-3'	-GGAACTCCTTAT-	72	-GTAGTATTCGAAGTTCC-3'	-GGAACTTCGAAT-
19	-GTAGTATAAGAAGTTCC-3'	-GGAACTTCTTAT-	73	-GTAGTATCCGAAGTTCC-3'	-GGAACTTCGGAT-
20	-GTAGTATAAGGTCTTCC-3'	-GGAAGACCTTAT-	74	-GTAGTATTCGGAGTTCC-3'	-GGAACTCCGAAT-
21	-GTAGTATAAGGTCATCC-3'	-GGATGACCTTAT-	75	-GTAGTATCCGGAGTTCC-3'	-GGAACTCCGGAT-
22	-GTAGTATAAGGTCGTCC-3'	-GGACGACCTTAT-	76	-GTAGTATTCGTTCTTCC-3'	-GGAAGAACGAAT-
23	-GTAGTATAAGATCTTCC-3'	-GGAAGATCTTAT-	77	-GTAGTATCCGTTCTTCC-3'	-GGAAGAACGGAT-
24	-GTAGTATAAGATCATCC-3'	-GGATGATCTTAT-	78	-GTAGTATTCGCTCTTCC-3'	-GGAAGAGCGAAT-
25	-GTAGTATAAGATCGTCC-3'	-GGACGATCTTAT-	79	-GTAGTATCCGCTCTTCC-3'	-GGAAGAGCGGAT-
26	-GTAGTATACGTAGTTCC-3'	-GGAACTACGTAT-	80	-GTAGTATTCGATCTTCC-3'	-GGAAGATCGAAT-
27	-GTAGTATACGCAGTTCC-3'	-GGAACTGCGTAT-	81	-GTAGTATCCGATCTTCC-3'	-GGAAGATCGGAT-
28	-GTAGTATACGAAGTTCC-3'	-GGAACTTCGTAT-	82	-GTAGTATTCGGTCTTCC-3'	-GGAAGACCGAAT-
29	-GTAGTATACGGAGTTCC-3'	-GGAACTCCGTAT-	83	-GTAGTATCCGGTCTTCC-3'	-GGAAGACCGGAT-
30	-GTAGTATACGTTCATCC-3'	-GGATGAACGTAT-	84	-GTAGTATTAGGTCATCC-3'	-GGATGACCTAAT-
31	-GTAGTATACGTTCGTCC-3'	-GGACGAACGTAT-	85	-GTAGTATCAGGTCATCC-3'	-GGATGACCTGAT-
32	-GTAGTATACGTTCTTCC-3'	-GGAAGAACGTAT-	86	-GTAGTATTAGATCATCC-3'	-GGATGATCTAAT-
33	-GTAGTATACGCTCATCC-3'	-GGATGAGCGTAT-	87	-GTAGTATCAGATCATCC-3'	-GGATGATCTGAT-
34	-GTAGTATACGCTCGTCC-3'	-GGACGAGCGTAT-	88	-GTAGTATTCGTTCATCC-3'	-GGATGAACGAAT-
35	-GTAGTATACGATCTTCC-3'	-GGAAGATCGTAT-	89	-GTAGTATCCGTTCATCC-3'	-GGATGAACGGAT-
36	-GTAGTATACGATCATCC-3'	-GGATGATCGTAT-	90	-GTAGTATTCGCTCATCC-3'	-GGATGAGCGAAT-
37	-GTAGTATACGATCGTCC-3'	-GGACGATCGTAT-	91	-GTAGTATCCGCTCATCC-3'	-GGATGAGCGGAT-
38	-GTAGTATACGGTCTTCC-3'	-GGAAGACCGTAT-	92	-GTAGTATTCGATCATCC-3'	-GGATGATCGAAT-
39	-GTAGTATACGGTCATCC-3'	-GGATGACCGTAT-	93	-GTAGTATCCGATCATCC-3'	-GGATGATCGGAT-
40	-GTAGTATACGGTCGTCC-3'	-GGACGACCGTAT-	94	-GTAGTATTCGGTCATCC-3'	-GGATGACCGAAT-
41	-GTAGTATCAGAAGCTCC-3'	-GGAGCTTCTGAT-	95	-GTAGTATCCGGTCATCC-3'	-GGATGACCGGAT-
42	-GTAGTATTAGGTCCTCC-3'	-GGAGGACCTAAT-	96	-GTAGTATTAGGTCGTCC-3'	-GGACGACCTAAT-
43	-GTAGTATCAGGTCCTCC-3'	-GGAGGACCTGAT-	97	-GTAGTATCAGGTCGTCC-3'	-GGACGACCTGAT-
44	-GTAGTATTAGATCCTCC-3'	-GGAGGATCTAAT-	98	-GTAGTATTAGATCGTCC-3'	-GGACGATCTAAT-
45	-GTAGTATCAGATCCTCC-3'	-GGAGGATCTGAT-	99	-GTAGTATCAGATCGTCC-3'	-GGACGATCTGAT-
46	-GTAGTATCCGTAGCTCC-3'	-GGAGCTACGGAT-	100	-GTAGTATTCGTTCGTCC-3'	-GGACGAACGAAT-
47	-GTAGTATTCGCAGCTCC-3'	-GGAGCTGCGAAT-	101	-GTAGTATCCGTTCGTCC-3'	-GGACGAACGGAT-
48	-GTAGTATTCGAAGCTCC-3'	-GGAGCTTCGAAT-	102	-GTAGTATTCGCTCGTCC-3'	-GGACGAGCGAAT-
49	-GTAGTATCCGAAGCTCC-3'	-GGAGCTTCGGAT-	103	-GTAGTATCCGCTCGTCC-3'	-GGACGAGCGGAT-
50	-GTAGTATTCGGAGCTCC-3'	-GGAGCTCCGAAT-	104	-GTAGTATTCGATCGTCC-3'	-GGACGATCGAAT-
51	-GTAGTATCCGGAGCTCC-3'	-GGAGCTCCGGAT-	105	-GTAGTATCCGATCGTCC-3'	-GGACGATCGGAT-
52	-GTAGTATTCGTTCCTCC-3'	-GGAGGAACGAAT-	106	-GTAGTATTCGGTCGTCC-3'	-GGACGACCGAAT-
53	-GTAGTATCCGTTCCTCC-3'	-GGAGGAACGGAT-	107	-GTAGTATCCGGTCGTCC-3'	-GGACGACCGGAT-
54	-GTAGTATTCGCTCCTCC-3'	-GGAGGAGCGAAT-	108	-GTAGTATACGCTCTTCC-3'	-GGAAGAGCGTAT-

* Entire probe sequence: 5'-GAA GAG TAT GGT TTC GTA TAC GTA AAA AAA AAA AAA AAA AAA AAA AXX XXX XXX XXX XXX XXX-3' (X represents tabulated probe sequence for clonotypes 1–108)

**Entire synthetic target sequence: Cy3–5'-CGG CCC GAA GTA CTG CTC GTA XXX XXX XXX XXX ACT ACT GGC ACA GAG ATA GAA AGC TGT CGG GTT CTT TTG G-3' (X represents tabulated synthetic target sequence for clonotypes 1–108)

**Figure 3:**
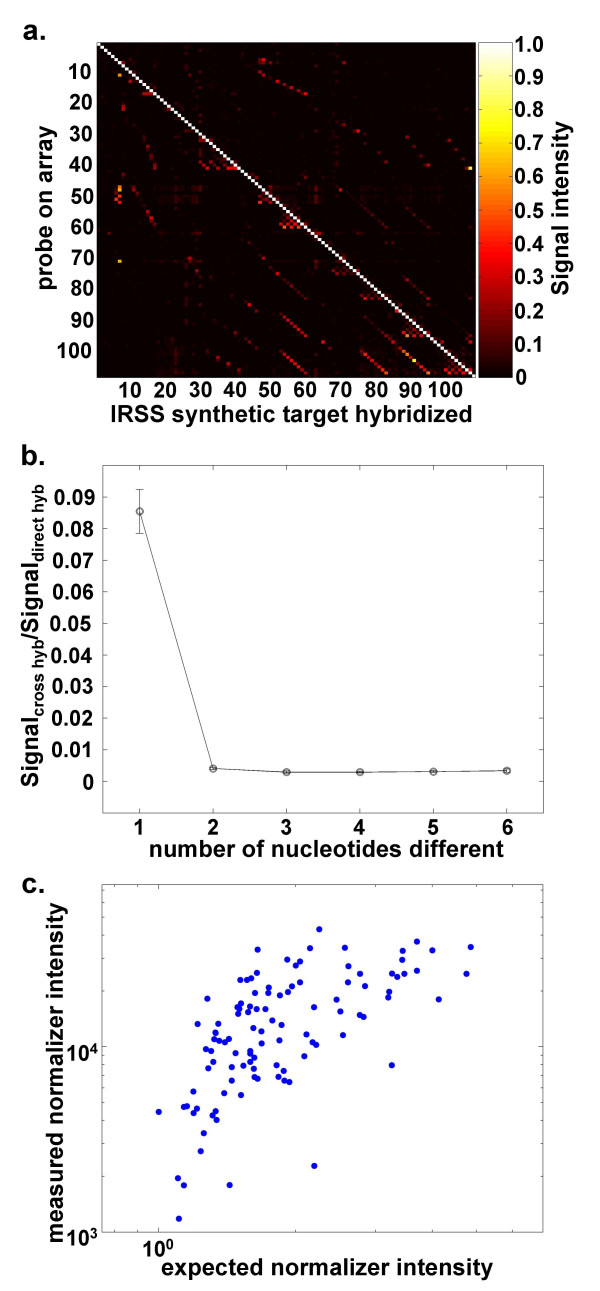
Analysis of each synthetic target hybridized independently to assess cross-hybridization. Panel A. Color map of independent hybridizations. Each column (x-axis) represents a slide to which a single synthetic target was hybridized (1–108). Each row (y-axis) represents a clonotype specific probe (1–108). Self-hybridization is normalized to 1. Panel B. The degree of cross hybridization is plotted against the number of nucleotide differences between probe:synthetic target pairs. Cross-hybridization arises from probe:synthetic target pairs that differ by one base. Panel C. Comparison of the measured normalizer intensity versus their expected profile. A good linear relationship is evident with R = 0.68, p < 0.001.

Mi=Ii+∑j≠iαij⋅Ij
 MathType@MTEF@5@5@+=feaafiart1ev1aaatCvAUfKttLearuWrP9MDH5MBPbIqV92AaeXatLxBI9gBaebbnrfifHhDYfgasaacH8akY=wiFfYdH8Gipec8Eeeu0xXdbba9frFj0=OqFfea0dXdd9vqai=hGuQ8kuc9pgc9s8qqaq=dirpe0xb9q8qiLsFr0=vr0=vr0dc8meaabaqaciaacaGaaeqabaqabeGadaaakeaacqWGnbqtdaWgaaWcbaGaemyAaKgabeaakiabg2da9iabdMeajnaaBaaaleaacqWGPbqAaeqaaOGaey4kaSYaaabuaeaaiiGacqWFXoqydaWgaaWcbaGaemyAaKMaemOAaOgabeaakiabgwSixlabdMeajnaaBaaaleaacqWGQbGAaeqaaaqaaiabdQgaQjabgcMi5kabdMgaPbqab0GaeyyeIuoaaaa@440D@

where α_*ij *_is a factor that measures the degree of cross hybridization from probe *j *and *I*_*i *_is the intensity of probe *i *when there is no cross hybridization (i.e. α_*ij *_= *0 for all j≠i*). To quantitatively evaluate cross hybridization (*A *= (α_*ij*_)), each of the 108 synthetic targets were independently hybridized to printed slides possessing 9 replicate arrays. Figure 2B illustrates such a hybridization. On each array (*k*), we expect all probe intensities to be zero (*I*_*i *_= 0 for all *i ≠ k*) except the probe (*k*) for which corresponding synthetic target has been introduced. Thus, α_*i*,*k *_was obtained for all *i, k*. This is represented by the color-map illustrated in Figure 3A. Although the specific hybridization of each synthetic target is clearly evident, variable degrees of cross hybridization existed between some synthetic target:probe combinations. We examined the dependence of cross hybridization (α_*ij*_) on the number of nucleotide differences between the possible synthetic target:probe pairs. Such pairs constituted 6.8% of all possible pairings (393/5886), and as expected cross hybridization primary arose from pairs that differed by a single nucleotide (Figure 3B). However, among all pairs that possess a single nucleotide difference, there is extensive heterogeneity in their cross hybridization, as evidenced by the large standard deviation.

When hybridizing known mixtures of synthetic target to the CDR3β array, we found that the resulting measured intensities did not correlate with the known input ratios. We have attributed this lack of linearity to differences in hybridization efficiency between the different IRSS encoding sequences, despite their high overall homlogy. Although the signal derived from a cohybridized *A. thaliana *synthetic target is useful for array quality control, using it to normalize potentially different amounts of support-bound probe between spots did not improve the overall correlation of reconstitution experiments (data not shown), lending support to the lack of linearity arising from differences in hybridization efficiency. Thus, we designed a dual hybridization format where the signals of an equimolar mixture of all 108 Cy3-labeled targets are used to normalize the Cy5-labeled CDR3β RT-PCR patient/subject signal (Figure 1). Since the normalizing mixture possesses all synthetic targets at an equal molar concentration, we would expect their intensity to be *I*_1 _= *I*_2 _= ... = *I*_108 _= *I*_0_, where *I*_0 _is a constant and their measured intensity to be:

Mnorm,i=∑j=1108αijIj=I0∑j=1108αij
 MathType@MTEF@5@5@+=feaafiart1ev1aaatCvAUfKttLearuWrP9MDH5MBPbIqV92AaeXatLxBI9gBaebbnrfifHhDYfgasaacH8akY=wiFfYdH8Gipec8Eeeu0xXdbba9frFj0=OqFfea0dXdd9vqai=hGuQ8kuc9pgc9s8qqaq=dirpe0xb9q8qiLsFr0=vr0=vr0dc8meaabaqaciaacaGaaeqabaqabeGadaaakeaacqWGnbqtdaWgaaWcbaGaeeOBa4Maee4Ba8MaeeOCaiNaeeyBa0MaeeilaWIaemyAaKgabeaakiabg2da9maaqahabaacciGae8xSde2aaSbaaSqaaiabdMgaPjabdQgaQbqabaGccqWGjbqsdaWgaaWcbaGaemOAaOgabeaaaeaacqWGQbGAcqGH9aqpcqaIXaqmaeaacqaIXaqmcqaIWaamcqaI4aaoa0GaeyyeIuoakiabg2da9iabdMeajnaaBaaaleaacqaIWaamaeqaaOWaaabCaeaacqWFXoqydaWgaaWcbaGaemyAaKMaemOAaOgabeaaaeaacqWGQbGAcqGH9aqpcqaIXaqmaeaacqaIXaqmcqaIWaamcqaI4aaoa0GaeyyeIuoaaaa@56AA@

In figure 3C the expected (∑j=1108αijI0
 MathType@MTEF@5@5@+=feaafiart1ev1aaatCvAUfKttLearuWrP9MDH5MBPbIqV92AaeXatLxBI9gBaebbnrfifHhDYfgasaacH8akY=wiFfYdH8Gipec8Eeeu0xXdbba9frFj0=OqFfea0dXdd9vqai=hGuQ8kuc9pgc9s8qqaq=dirpe0xb9q8qiLsFr0=vr0=vr0dc8meaabaqaciaacaGaaeqabaqabeGadaaakeaadaaeWbqaaGGaciab=f7aHnaaBaaaleaacqWGPbqAcqWGQbGAaeqaaaqaaiabdQgaQjabg2da9iabigdaXaqaaiabigdaXiabicdaWiabiIda4aqdcqGHris5aOGaemysaK0aaSbaaSqaaiabicdaWaqabaaaaa@3BDB@) and measured intensities for all synthetic targets are plotted where a linear relationship (r = 0.68, p < 0.001) is observed.

### Evaluation of assay quantitativeness through reconstitution studies

To evaluate the ability of the assay to accurately identify and quantify different CDR3β sequences within a complex mixture, reconstitution studies were performed. To avoid purchasing two sets of synthetic targets, these validation experiments were conducted in a single color format (Cy3), with the normalizing equimolar mixture of all 108 synthetic targets hybridized to separate slides (n = 5). Twenty six unique test mixtures were prepared where 6 to 30 different synthetic targets (mean 16.6 +/- 7.4) were introduced at percentages of 0.5% to 48.82%. The test mixtures possessed 1 to 5 total picomoles of synthetic target. Within the 26 mixtures, each of the 108 clonotypes was measured at least 3 times at 3 different input percentages. Each test mixture was analyzed through hybridization to 9 to 18 replicates arrays.

**Figure 4:**
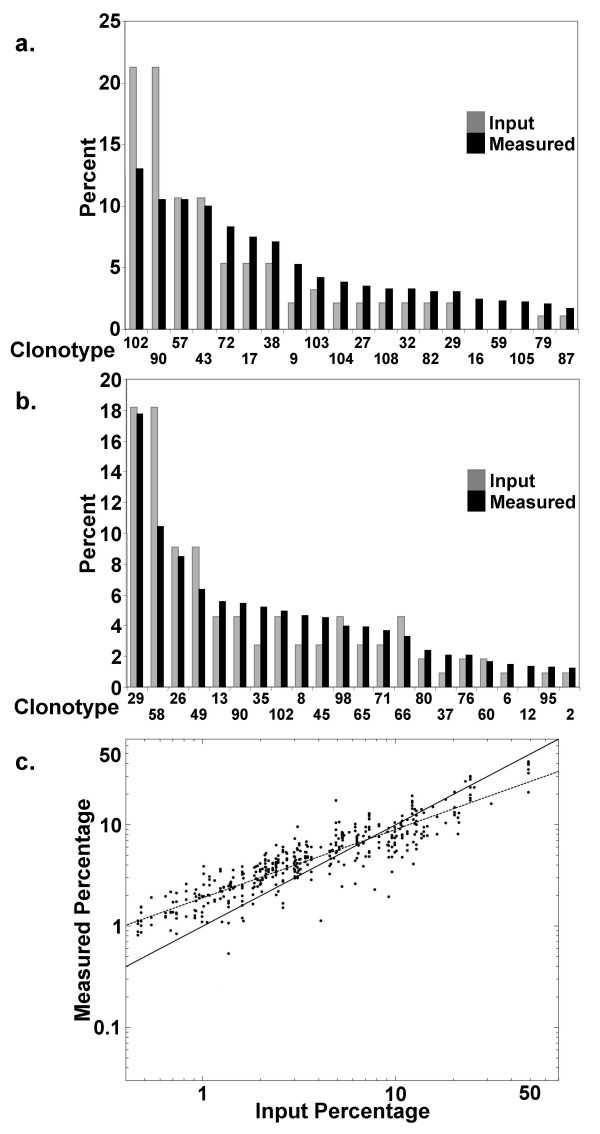
Assessment of assay performance through oligonucleotide reconstitution studies. Panels A and B: Known input percentages are indicated (grey, total 100%), measured percentages are indicated in black. Clonotype number is indicated on *x-axis*, arranged by descending measured percentage. All measured percentages are shown to the level of the lowest input percentage. Panel C: Summary plot of the 26 reconstitution mixes representing all 108 clonotypes. Dotted line shows correlation between input and measured percentages (R = 0.90, p < 0.0001, log_10_M = 10^0.68 ^*log_10_I + 0.28). A 45° reference line (solid) is shown.

In each of the 26 mixes, the actual clonotype abundances were closely captured by measured abundances. The results of representative mixes are shown in Figure 4A–B. We found the system sensitive to input percentages as low as 0.5%. The data from all 26 mixes was collectively analyzed (Figure 4C). Despite some under estimation of high input percentages and some over estimation of low input percentages, good agreement between known input percentages and measured percentages was observed (R = 0.90, p < 0.0001). Overall, we observed intra and inter-slide variances of 3.0 and 3.7%, respectively. The data were examined to determine if any probe consistently over or under-measured its corresponding input percentage and no biases were detected (data not shown). As reflected in Figures 4A and 4B reliable measured percentages were observed within mixes down to the 2–3% input level, below which false-positive calls were detected. Therefore the false discovery rate (FDR)[18] which was defined as fraction of clonotypes that reached the detection limit that were not introduced into the test mixture, at different detection sensitivities was examined. At a detection sensitivity of 4% or higher, the FDR was equal to 0. At detection sensitivities between 3% and 1%, the FDR ranged from 2.3% to 13%, respectively.

### Analysis of M158–66/HLA-A2.1-specific CD8 T-cell cultures by traditional clonotyping and array analysis

To validate the array in T-cell repertoire analysis, it was used to analyze human recall responses to influenza M1_58–66 _restricted by HLA-A2. Three human subjects were analyzed (designated A, B, and C in Tables 2, 3, and 4, respectively). In each case triplicate T-cell cultures were propagated (designated 1, 2, and 3 for each subject) then analyzed by microarray and traditional clonotyping. Short term recall cultures are a standard assay for increasing the frequency of antigen-specific clonotypes in the sample. This occurs because the specific T cells expand and some of the non-specific T cells die out in the cultures. Cultures are performed in triplicate to control for the variation that arises in a complex biological response *in vitro*.

**Table 2:** Comparison of array based clonotyping to cloning and sequencing of PCR products: Subject A

	**Array analysis**					**Cloning/sequencing**				
**Clonotype**	**1**	**2**	**3**	**Mean**	**(SD)**	**1**	**2**	**3**	**Mean**	**(SD)**

50	38	11	27	25.4	(+/-13.6)	20	0	0	6.7	(+/-11.5)
41	2	16	32	16.5	(+/-14.8)	20	27	40	28.9	(+/-10.2)
51	12	37	0	16.3	(+/-18.7)	10	40	0	16.7	(+/-20.8)
58	8	3	16	9.0	(+/-6.3)	0	0	0	0	(+/-0)
4	6	8	4	5.7	(+/-2.1)	10	7	0	5.6	(+/-5.1)
48	7	0	7	4.7	(+/-4.1)	10	0	27	12.2	(+/-13.5)
42	6	7	0	4.1	(+/-3.6)	10	13	0	7.8	(+/-6.9)
13	2	8	0	3.3	(+/-4.0)	0	0	7	2.2	(+/-3.8)
8	3	2	4	3.1	(+/-0.8)	0	7	0	2.2	(+/-3.8)
5	0	0	9	2.8	(+/-4.9)	0	0	27	8.9	(+/-15.4)
1	8	0	0	2.6	(+/-4.5)	20	0	0	6.7	(+/-11.5)
3	6	0	0	1.9	(+/-3.2)	0	0	0	0	(+/-0)
90	0	4	0	1.5	(+/-2.6)	0	0	0	0	(+/-0)
82	1	1	1	1.0	(+/-0)	0	0	0	0	(+/-0)
17	0	0	1	0.4	(+/-0.7)	0	0	0	0	(+/-0)
74	1	0	0	0.4	(+/-0.6)	0	0	0	0	(+/-0)
49	0	1	0	0.4	(+/-0.6)	0	0	0	0	(+/-0)
52	0	1	0	0.4	(+/-0.6)	0	0	0	0	(+/-0)
75	0	1	0	0.4	(+/-0.6)	0	0	0	0	(+/-0)
91	0	0	0	0.0	(+/-0)	0	7	0	2.2	(+/-3.8)

**Table 3:** Comparison of array based clonotyping to cloning and sequencing of PCR products: Subject B

	**Array analysis**					**Cloning/sequencing**				
**Clonotype**	**1**	**2**	**3**	**Mean**	**(SD)**	**1**	**2**	**3**	**Mean**	**(SD)**

50	61	45	70	58.6	(+/-12.5)	46	24	0	23.2	(+/-23)
4	11	25	11	15.5	(+/-7.8)	30	53	36	39.8	(+/-11.7)
13	0	12	7	6.3	(+/-5.9)	3	14	11	9.1	(+/-5.4)
64	12	3	0	5.0	(+/-6.3)	3	1	0	1.3	(+/-1.6)
94	7	8	0	4.9	(+/-4.3)	6	7	1	4.9	(+/-3.1)
83	0	0	5	1.7	(+/-2.9)	0	0	0	0	(+/-0)
11	5	0	0	1.7	(+/-2.9)	5	0	0	1.8	(+/-3.2)
1	0	2	2	1.3	(+/-1.1)	2	0	0	0.5	(+/-0.9)
93	0	0	3	1.0	(+/-1.7)	0	0	0	0	(+/-0)
53	2	0	0	0.7	(+/-1.2)	0	0	0	0	(+/-0)
47	0	0	1	0.3	(+/-0.6)	0	0	50	16.7	(+/-28.9)
44	0	0	1	0.3	(+/-0.6)	0	0	0	0	(+/-0)
49	1	0	0	0.3	(+/-0.6)	2	1	0	0.7	(+/-0.8)
107	1	0	0	0.3	(+/-0.6)	1	0	0	0.3	(+/-0.5)
2	0	1	0	0.3	(+/-0.6)	1	0	0	0.3	(+/-0.5)
51	0	1	0	0.3	(+/-0.6)	1	0	0	0.3	(+/-0.5)
17	0	1	0	0.3	(+/-0.6)	0	0	0	0	(+/-0)
30	0	1	0	0.3	(+/-0.6)	0	0	0	0	(+/-0)
41	0	1	0	0.3	(+/-0.6)	0	0	0	0	(+/-0)
42	0	1	0	0.3	(+/-0.6)	0	0	0	0	(+/-0)
84	0	0	0	0	(+/-0)	0	0	2	0.7	(+/-1.2)
5	0	0	0	0	(+/-0)	0	1	0	0.4	(+/-0.8)

**Table 4:** Comparison of array based clonotyping to cloning and sequencing of PCR products: Subject C

	**Array analysis**					**Cloning/sequencing**				
**Clonotype**	**1**	**2**	**3**	**Mean**	**(SD)**	**1**	**2**	**3**	**Mean**	**(SD)**

50	9	23	16	16.1	(+/-7.1)	4	1	0	1.5	(+/-1.8)
70	26	12	0	12.6	(+/-12.9)	61	2	0	20.9	(+/-34.5)
51	19	1	4	8.1	(+/-9.5)	21	0	9	10.0	(+/-10.8)
2	11	11	0	7.3	(+/-6.3)	10	33	57	33.1	(+/-23.5)
8	0	21	0	7.0	(+/-12.1)	0	56	0	18.7	(+/-32.4)
60	5	0	11	5.3	(+/-5.5)	0	0	0	0	(+/-0)
20	0	0	14	4.8	(+/-8.2)	0	0	0	0	(+/-0)
6	3	2	5	3.5	(+/-1.8)	0	2	22	7.9	(+/-12.0)
58	0	2	8	3.2	(+/-4.0)	0	0	0	0	(+/-0)
47	3	0	5	2.8	(+/-2.8)	0	0	0	0	(+/-0)
88	0	0	8	2.6	(+/-4.4)	0	0	0	0	(+/-0)
106	0	1	7	2.5	(+/-3.6)	0	0	0	0	(+/-0)
18	0	7	0	2.3	(+/-4.0)	0	0	0	0	(+/-0)
71	2	1	3	2.1	(+/-1.2)	0	0	0	0	(+/-0)
82	0	0	5	1.8	(+/-3.2)	0	0	0	0	(+/-0)
61	0	1	4	1.8	(+/-2.3)	0	0	0	0	(+/-0)
86	0	5	0	1.7	(+/-2.9)	0	0	0	0	(+/-0)
7	2	3	0	1.7	(+/-1.5)	0	0	0	0	(+/-0)
94	0	0	4	1.5	(+/-2.5)	0	0	0	0	(+/-0)
21	0	4	0	1.3	(+/-2.3)	0	0	0	0	(+/-0)
49	4	0	0	1.3	(+/-2.3)	0	0	0	0	(+/-0)
26	4	0	0	1.3	(+/-2.3)	0	0	0	0	(+/-0)
42	4	0	0	1.3	(+/-2.3)	0	0	0	0	(+/-0)
67	4	0	0	1.3	(+/-2.3)	0	0	0	0	(+/-0)
74	0	0	3	1.1	(+/-1.9)	0	0	0	0	(+/-0)
52	0	3	0	1.0	(+/-1.7)	0	1	0	0.3	(+/-0.5)
84	0	3	0	1.0	(+/-1.7)	0	0	0	0	(+/-0)
75	3	0	0	1.0	(+/-1.7)	0	0	0	0	(+/-0)
55	2	0	0	0.7	(+/-1.1)	0	0	0	0	(+/-0)
1	0	0	0	0	(+/-0)	4	4	10	5.7	(+/-3.5)
23	0	0	0	0	(+/-0)	0	0	2	0.7	(+/-1.3)
69	0	0	0	0	(+/-0)	1	0	0	0.4	(+/-0.7)
90	0	0	0	0	(+/-0)	0	0	1	0.4	(+/-0.6)
4	0	0	0	0	(+/-0)	0	1	0	0.3	(+/-0.5)

RNA extracted from each culture was reverse transcribed, and amplified with forward and reverse primers targeting the VB17 and JB2.7 segments, respectively. In the case of the microarray analysis, the product was amplified with Cy5 labeled JB2.7 primer, purified and hybridized, as illustrated in Figure 1. With the traditional approach, unlabeled product was cloned into a plasmid vector, transformed into *Escherichia coli*, plated, and 188 colonies per culture (564 colonies per subject) were sequenced to establish clonal diversity.

In the reconstitution studies, we determined that at a sensitivity of detection ≥ 4% the FDR was equal to 0. When comparing the microarray data to traditional clonotyping under these strictest standards (ie a clonotype must be detected within at least 1 culture at ≥ 4% abundance in the array analysis), we observe that on average, the array detects slightly more clonotypes than does cloning and sequencing (1.1-fold more: 42 versus 37; subject A: 13 versus 12; subject B: 7 versus 14; subject C: 22 versus 12). In subject A, the array detected 2 clonotypes not observed by cloning, while cloning identified 1 clonotype not observed by the array. In subject B, the array detected 1 clonotype not observed by cloning, while cloning detected 8 clonotypes not observed by the array. In subject C, the array detected 16 clonotypes not observed by cloning, while cloning identified 6 clonotypes not observed by the array.

If the data is examined using a 1% sensitivity of detection (FDR = 13%) the total number of clonotypes detected the by array analysis among the three subjects increases from 42 to 68. This increased sensitivity of 26 additional clonotypes would be predicted to include approximately 3 false positives. At this stringency, the array detected nearly twice as many clonotypes than did cloning/sequencing (1.8-fold more: 68 versus 37; culture A: 19 versus 11; culture B: 20 versus 14; culture C: 29 versus 12). In analysis A, the array detected 9 clonotypes not observed by cloning, while cloning identified 1 clonotype not observed by the array. In analysis B, the array detected 8 clonotypes not observed by cloning, while cloning detected 2 clonotypes not observed by the array. In analysis C, the array detected 22 clonotypes not observed by cloning, while cloning identified 5 clonotypes not observed by the array.

Among subjects A, B and C there were respectively 10, 12, and 7 clonotypes commonly detected by the array and cloning/sequencing. When evaluating the rank order of clonotype abundance determined by each method, the approaches are not significantly different (Wilcoxon rank-sum test, p > 0.05).

## Discussion

In this report, we describe a spotted oligonucleotide microarray application for the investigation of cytotoxic memory T-cell repertoires against influenza matrix protein, M1_58–66 _in HLA-A2 individuals. This expandable approach specifically identifies 108 highly related CDR3β sequences that encode the IRSS core amino acid motif and accurately quantifies their relative abundances within a complex mixture. The design, development, and use of this assay depended on the use of novel strategies to control for array fabrication variables and vendor oligonucleotide synthesis quality, as well as to normalize differences in hybridization efficiencies between the closely related target sequences.

The microarray application described here is unlike most array assays. Typically, the arrayed probes represent up to tens of thousands of independent parallel assays, where each probe measures the relative abundance of a single distinct gene among the samples being compared. In this application each of the 108 CDR3β-specific probes measure the contribution of individual clonotypes within a single sample to define the T-cell repertoire. In order for the repertoire to be accurately measured, it is paramount to control any array fabrication and/or oligonucleotide probe variables that might lead to erroneously high or low signal intensities at any given address on the array. The susceptibility of spotted microarrays to fabrication-based quality control issues is well documented [19-22]. When array imaging is conducted with confocal laser scanners possessing narrow bandwidths, fluorescein is compatible with the commonly used cyanine dyes. Thus, we typically tag cDNA probes during amplification using fluorescein-labeled oligonucleotide primers [23,24]. However, fluorescently modifying oligonucleotide probe sets is cost prohibitive, therefore, we co-spot a fluorescein-labeled "tracking" oligonucleotide at low molarity with the target-specific probes [15] when printing oligonucleotide arrays. In either case, labeling the array with a third fluorophore, enables array quality control prior to hybridization of potentially precious samples to substandard arrays.

As useful as the tracking oligonucleotide is, it is not fully informative in controlling oligonucleotide synthesis. The novel dual specificity probe design described here possesses a 5'-region specific for the *A. thaliana *LTP4 gene intended to facilitate evaluation of probe synthesis. This region is synthesized after the 3' CDR3β-specific region, thus as shown in Figure 2A, a single hybridization with *A. thaliana *LTP4 synthetic target can provide control of oligonucleotide synthesis for the entire probe set. Thus, using the *A. thaliana *LTP4 signal intensity it is possible to show relative equality of the probes across the array, a necessary prerequisite to specificity testing of each probe with the synthetic targets. An unexplored and more costly alternative to ensure immobilization of full-length probes would be to print 5' amine modified oligonucleotides onto slides coated with N-hydroxysuccinimide (NHS) ester reactive groups. We have also empirically determined that the 63-mer probe design yields better signal intensities upon hybridization of synthetic targets compared to 17-mer probes encompassing only the CDR3β, despite the high surface density that can be achieved by printing the short probe (data not shown). This effect is attributable to the possibility that labeled target molecules may have less steric interference and greater access to the longer probes [25-27]. Thus, our probe design provides a means of synthesis quality control, possesses a short CDR3β-specific region for high specificity, yet is of sufficient length to generate robust hybridization signals.

In typical array-based gene expression or genotyping applications, it is desirable that all the probes on the array possess similar hybridization characteristics, however, minor performance differences are tolerable since comparisons of different samples are made across a single probe. In this application, single samples are quantitatively analyzed over multiple probes, making it necessary to account for differences in probe efficiencies. Having quantified the performance of probes for the 108 IRSS encoding CDR3β clonotypes it is theoretically possible to mathematically correct differences in hybridization efficiency. In practice we found that this approach is cumbersome. Although the array performs in a predictable manner, the exact degree of correction for each probe is dependent on experimental conditions and array fabrication. Thus, proper mathematical correction would require implementing sophisticated algorithms to correct for the inter-experiment differences. This is neither realistic nor convenient. In view of the situation, we have instead designed a practical approach that employs hybridization of the equimolar mixture Cy3-labeled synthetic targets to allow normalization of differences in hybridization efficiencies between probes.

Spectratyping and clonotyping have been the mainstays of analyzing TCR diversity for over a decade, however, in addition to the strategy described here, other array-based strategies have been employed. Ogle et al., [28] directly measured lymphocyte receptor diversity by utilizing the cross-hybridization patterns of T or B cell transcripts to Affymetrix GeneChips. More recently, Bonarius et al., [29] interrogated TCR diversity by first hybridizing CDR3-specific "annealers" to amplified product, which in turn were ligated to a random hexamer array. While this elegant approach provides a measurement of the overall T-cell repertoire, it has not been shown able to quantitatively resolve clonotypes differing by a single nucleotide.

## Conclusion

We have validated this new array strategy through reconstitution studies, using known mixtures of synthetic target (Figure 4), as well as through comparison to traditional cloning and sequencing of VB17-JB2.7 PCR product (Tables 2, 3, 4). These studies show that array-based clonotyping is a reliable, efficient, and cost effective approach that is at least as informative as traditional clonotyping. While unmodified 63-mer probes are very affordable, the set of labeled synthetic targets for normalization are relatively expensive. However, the reagent costs required to set up this type of array-based genotyping system can be distributed over thousands of analyses. Thus, in terms of both materials and labor, the cost of clonotyping by array is less that than cloning and sequencing. The array based method is also more direct in that there are fewer steps between RT-PCR and data acquisition, thus exhaustive cloning and its potential biases are avoided. As with any genotyping assay, one must have knowledge of the sequences being assayed, therefore a practical limitation of the approach is the requirement for a priori knowledge of the TCR usage of the T-cell response under analysis. Once this has been established, the array based approach allows for much higher through-put making more feasible population based studies of the human immune response to influenza A. These efforts lay the foundation for expanding the clonotyping array to cover additional motifs, including those VB17-JB2.7 rearrangements encoding IRSA, VRSS, MRSA, and SRSS, relevant to monitoring memory responses to influenza A. The approach used here is broadly applicable to situations where complex mixtures of highly homologous sequences need to be quantitatively resolved. Examples of such applications include HLA typing, viral or bacterial genotyping and characterization microbial species within environmental samples.

## Methods

### Oligonucleotide probes and synthetic targets

Oligonucleotide probes (63-mers) specific for 108 different CDR3 motifs encoding the core amino acids IRSS were designed with the assistance of the Oligo 6.0 software (Molecular Biology Insights, Inc., Cascade, CO) (Table 1). Probe sequences were evaluated for dimer formation, interfering secondary structure, GC content and melting temperature. The probes were designed with a 23 nucleotide 5'-region specific for the *A. thaliana *LTP4 (lipid transfer protein 4) gene; a 23 nucleotide polyA spacer, and a 17 nucleotide IRSS-specific region (sense strand) placed at the 3'-end of the probe.

Synthetic targets consisted of set of 108 5'-Cy3-labeled 73-mers (anti-sense strand, Table 1). These positive controls were necessary for testing probe specificity as well as for normalization. They precisely mimic the 73 bp RT-PCR product generated by the BV17 and BJ2.7-specific oligonucleotide primers used for clonotyping. All probes and synthetic targets were supplied by Integrated DNA Technologies (Coralville, IA) and their concentrations were measured on a UV spectrophotometer (SpectraMax Plus 384, Molecular Devices, Sunnyvale, CA).

### Array fabrication

Oligonucleotide probes were reconstituted at a final concentration of 40 μM in printing buffer (1.5 M betaine, 3% DMSO, 0.5 uM tracking oligonucleotide). As previously described [15], the tracking oligonucleotide [5'-(FITC)-ATGAAGAAACTATATACATCTTATGGCACTTATGGATTTTTACATCAGATAAAAATCAATAACCCGACCC-3'], based upon the *Staphylococcus aureus *response regulator trap, is fluorescein-tagged and co-spotted at a 1:80 molar disadvantage relative to target-specific oligonucleotides. The tracking oligonucleotide does not interfere with the hybridization of targets to target-specific probes, but enables array visualization and quality control prior to hybridization [15].

The oligonucleotide probes were printed onto poly-L-lysine coated slides using a GeneMachines Omnigrid arrayer (San Carlos, CA) and Telechem International SMP3 pins (Sunnyvale, CA) at 40% humidity and 24°C. To control pin contact force and duration, the instrument was set with the following Z motion parameters, velocity: 7 cm/sec, acceleration: 100 cm/sec2, deceleration: 100 cm/sec2. After printing, oligonucleotides were UV cross-linked to slides and processed using the previously described nonaqueous blocking protocol [30]. Prehybridization fluorescein images were collected using a ScanArray 5000 (GSI Lumonics, Billerica, MA) to assess overall printing fidelity and probe deposition. Nine replicate arrays were printed on each glass slide.

### Generation of M158–66/HLA-A2.1-specific CD8 T-cell cultures

Influenza M1_58–66_/HLA-A2.1-specific CD8 T-cell cultures were generated by stimulating 2 × 10^6 ^cells at 1 × 10^6^/1 ml cell concentration, 1 uM M1_58–66 _peptide final concentration, and 10 unit/ml human recombinant IL2, in a 13 ml round bottom tube. The culture was carried for 2 weeks with adding IL2 every 3 days and re-stimulating after the first week. Re-stimulation used irradiated (3000 RAD) autologous PBMC pulsed with peptide for 3 hours at a 1/1 ratio with the cultured cells. Cultures typically possessed approximately 5 × 10^5 ^cells at the end of two weeks. All cultures were performed in triplicate.

### RNA isolation, cDNA synthesis and spectratyping

RNA was isolated from cultured cells using Dynabeads mRNA direct kit as instructed (Invitrogen, Carlsbad, CA) and cDNA synthesis, CDR3β spectratyping, primer sequences and electrophoretic conditions have been previously described [31]. Briefly, cDNA samples were amplified by PCR using VB17-specific forward primer and J-specific carboxyfluorescein-labeled reverse primers for 30–32 cycles (non-saturating PCR reactions to minimize possibility of changing distribution of sequences). 10 ul of the amplified product was separated by electrophoresis through 5 M urea/5% polyacrylamide sequencing gels. Gels were imaged with a FluorImager 595 fluorescence detection system (Molecular Dynamics, Piscataway, NJ). The 150 base PCR fragment was cut out of the gel, DNA was extracted from the gel piece using QIAEX II DNA extraction kit as instructed (QIAgen, Valencia, CA), 10 ul of the extracted DNA was re-amplified for 3 cycles in a 20 ul reaction using unlabelled constant primer. Ligation and transformation was done using pCR4-TOPO kit (Invitrogen). Glycerol stocks of bacterial colonies were sequenced by a commercial vendor (Genewiz, North Brunswick, NJ). CDR3 junction sequences were identified and analyzed using Finch TV (htpp://www.geospiza.com/finchtv/). Analysis of VB17 and J region flanking sequences indicated <0.5% divergence from the genomic sequences, which can be attributed to reverse transcriptase and AmpliTaq DNA polymerase infidelity. Only clonotypes containing a VB17 CDR3 that were ten amino acid residues in length were subject to statistical analysis.

### Array sample and hybridization

VB17 repertoire analysis by microarray utilized the same M1-specific CD8 T-cell cDNA samples described above. One microliter of cDNA was amplified with unlabeled VB17-specific forward [5'-CCAAAAGAACCCGACAGCTTTC-3'] and Cy5 labeled J2.7-specific reverse [5'-(Cy5)-CGGCCCGAAGTACTGCTCG-3'] primers in a 40 μl non-saturating PCR reaction using a commercial PCR Master Mix (Promega, Madison, WI), possessing 25 u/ml Taq, 200 μM dNTPs and 1.5 mM MgCl2. Forward and reverse primers were added for a final concentration of 0.25 μM. Thirty-two cycles of PCR were performed (94°C, 30 seconds; 55°C, 30 seconds; 72°C, 30 seconds) in a Perkin-Elmer 9700 thermal cycler. PCRs were terminated after a 7-minute extension at 72°C and analyzed by 10% TBE gel electrophoresis to confirm presence of a single robust 73 bp product. To each PCR product, 10.9 μl IRSS normalizing synthetic target mix was added. This consisted of an equimolar mix of all 108 synthetic targets at a final concentration of 0.1 μM each. The labeled cDNA was purified with Centri-Sep columns (Princeton Separations, Adelphia, NJ) and dried to completion. The sample was then reconstituted in 27 μl of hybridization buffer (5.2× Denhardt's solution, 6.3× SSC, 0.5% SDS and 52% formamide), heated at 95°C for 2 minutes and centrifuged for 6 minutes at 14,000 rpm. Twenty-three microliters of sample was applied to the array and covered with a glass cover slip. The array was loaded into a Corning hybridization chamber and incubated at 42°C for 16–18 hours. The cover slips were removed by briefly immersing the arrays in 2× SSC and 0.1% SDS. The arrays were then washed in 2× SSC, 1× SSC, 0.2× SSC and 0.05× SSC for 4 minutes, 1.5 minutes, 1.5 minutes and 10 seconds respectively. The slides were spun dry at 100 rfu for 10 minutes at room temperature. Cy3, Cy5 and FITC image files were generated by analyzing the arrays with a ScanArray 5000 (GSI Luminonics, Billerica, MA) confocal laser scanner.

### Microarray data acquisition, filtering, and analysis

Array images were processed, analyzed, and subjected to quality-dependent filtering using the Matarray software as previously described [32,33]. The analysis first involved measurement of the "unknown sample" signal intensity for each probe. In the case of human T-cell culture samples, this was measurement of the Cy5 signal intensity derived from the hybridized CDR3β RT-PCR product. In the case of "reconstituted unknown samples" this was measurement of Cy3 signal intensity derived from known mixtures of synthetic targets. Signal intensities for the equimolar mix of 108 synthetic targets was also determined. This allowed for normalization of the hybridization efficiency differences between different CDR3β sequences and their corresponding probes, thereby making it possible to determine the relative abundances of the different clonotypes within the sample. When conducting reconstitution experiments with oligonucleotide mixtures, the unknown and equimolar normalizing mix were hybridized to separate arrays since they both utilized the same fluorochrome (Cy3). When analyzing human subject samples, the unknown and normalizing synthetic target mix were cohybridized since they were tagged with different cyanine dyes, Cy5 and Cy3, respectively.

Each analysis was conducted on duplicate glass slides, since each slide possessed 9 arrays, a total of 18 replicates were performed. After raw signal intensity values were collected, the mean and CV (coefficient of variance) were determined for CDR3β and normalizer signals for each of the 108 probes. Data points possessing a quality score of *q*_*com *_= 0 were discarded. These primarily arose from signals that were not resolvable from background noise. For probes where the CV exceeded the median of all 108 CVs + 2SD (standard deviations), the 2 most outlying data points were discarded and the mean and CV were recalculated. The normalized ratio for each of the 108 probes was then determined by dividing the CDR3β signal intensity by the normalizer signal intensity for each probe, as follows:

Normalized log_10 _Ratio = log_10 _CDR3β (probe 1–108) – log_10 _normalizer (probe 1–108)

In any single sample, the majority of possible sequences were not present. When analyzing the normalized intensity data as a log ratio distribution, 2 subgroups were evident. One group, typically consisted of approximately 80% of the probes; these exhibited very low basal level intensity. In contrast, the other group possessed significantly higher intensity, which often, as expected [13,14], exhibited a power-law distribution. For analysis, this group was defined as those probes exhibiting intensities greater than 2SD above the mean intensity of all probes. Thus, to determine the relative abundances of the different CDR3β sequences present in the sample, group 1 probe normalized intensities were set to 0. The normalized intensities of the second group were divided by their sum so that each CDR3β signal could be expressed as a percentage of the total.

## Competing interests

The author(s) declares that there are no competing interests.

## Authors' contributions

WX designed the data analysis strategy and participated in drafting of manuscript. SJ participated in designing data analysis strategy and carried out all array data analysis. LM designed array probes and carried out array development and validation experiments. MY and YN conducted clonotyping analysis. JG participated in the design of the study. MJH conceived of the study, participated in its design and coordination, and drafted the manuscript. All authors read and approved the final manuscript.

## References

[B1] Davis MM, Bjorkman PJ (1988). T-cell antigen receptor genes and T-cell recognition. Nature.

[B2] Turner SJ, Doherty PC, McCluskey J, Rossjohn J (2006). Structural determinants of T-cell receptor bias in immunity. Nat Rev Immunol.

[B3] Lehner PJ, Wang EC, Moss PA, Williams S, Platt K, Friedman SM, Bell JI, Borysiewicz LK (1995). Human HLA-A0201-restricted cytotoxic T lymphocyte recognition of influenza A is dominated by T cells bearing the V beta 17 gene segment. J Exp Med.

[B4] Moss DJ, Burrows SR, Baxter GD, Lavin MF (1991). T cell-T cell killing is induced by specific epitopes: evidence for an apoptotic mechanism. J Exp Med.

[B5] Arstila TP, Casrouge A, Baron V, Even J, Kanellopoulos J, Kourilsky P (1999). A direct estimate of the human alphabeta T cell receptor diversity. Science.

[B6] Arstila TP, Casrouge A, Baron V, Even J, Kanellopoulos J, Kourilsky P (2000). Diversity of human alpha beta T cell receptors. Science.

[B7] Maslanka K, Piatek T, Gorski J, Yassai M (1995). Molecular analysis of T cell repertoires. Spectratypes generated by multiplex polymerase chain reaction and evaluated by radioactivity or fluorescence. Hum Immunol.

[B8] Naumov YN, Hogan KT, Naumova EN, Pagel JT, Gorski J (1998). A class I MHC-restricted recall response to a viral peptide is highly polyclonal despite stringent CDR3 selection: implications for establishing memory T cell repertoires in "real-world" conditions. J Immunol.

[B9] Gorski J, Yassai M, Zhu X, Kissela B, Kissella B, Keever C, Flomenberg N (1994). Circulating T cell repertoire complexity in normal individuals and bone marrow recipients analyzed by CDR3 size spectratyping. Correlation with immune status. J Immunol.

[B10] Gorski J, Piatek T, Yassai M, Maslanka K (1995). Improvements in repertoire analysis by CDR3 size spectratyping. Bifamily PCR. Ann N Y Acad Sci.

[B11] Douek DC, Betts MR, Brenchley JM, Hill BJ, Ambrozak DR, Ngai KL, Karandikar NJ, Casazza JP, Koup RA (2002). A novel approach to the analysis of specificity, clonality, and frequency of HIV-specific T cell responses reveals a potential mechanism for control of viral escape. J Immunol.

[B12] Naumov YN, Naumova EN, Gorski J (1996). CD4+ and CD8+ circulating alpha/beta T-cell repertoires are equally complex and are characterized by different levels of steady-state TCR expression. Hum Immunol.

[B13] Naumov YN, Naumova EN, Clute SC, Watkin LB, Kota K, Gorski J, Selin LK (2006). Complex T cell memory repertoires participate in recall responses at extremes of antigenic load. J Immunol.

[B14] Naumov YN, Naumova EN, Hogan KT, Selin LK, Gorski J (2003). A fractal clonotype distribution in the CD8+ memory T cell repertoire could optimize potential for immune responses. J Immunol.

[B15] Hessner MJ, Singh VK, Wang X, Khan S, Tschannen MR, Zahrt TC (2004). Utilization of a labeled tracking oligonucleotide for visualization and quality control of spotted 70-mer arrays. BMC Genomics.

[B16] Hessner MJ, Baxter-Lowe LA (1992). Characterization of novel HLA-DPB1 alleles by oligotyping and nucleotide sequencing. Tissue Antigens.

[B17] Baxter-Lowe LA, Hunter JB, Casper JT, Gorski J (1989). HLA gene amplification and hybridization analysis of polymorphism. HLA matching for bone marrow transplantation of a patient with HLA-deficient severe combined immunodeficiency syndrome. J Clin Invest.

[B18] Storey JD (2002). A direct approach to false discovery rates. Journal of the Royal Statistical Society.

[B19] Shi L, Tong W, Goodsaid F, Frueh FW, Fang H, Han T, Fuscoe JC, Casciano DA (2004). QA/QC: challenges and pitfalls facing the microarray community and regulatory agencies. Expert Rev Mol Diagn.

[B20] Irizarry RA, Warren D, Spencer F, Kim IF, Biswal S, Frank BC, Gabrielson E, Garcia JG, Geoghegan J, Germino G, Griffin C, Hilmer SC, Hoffman E, Jedlicka AE, Kawasaki E, Martinez-Murillo F, Morsberger L, Lee H, Petersen D, Quackenbush J, Scott A, Wilson M, Yang Y, Ye SQ, Yu W (2005). Multiple-laboratory comparison of microarray platforms. Nat Methods.

[B21] Larkin JE, Frank BC, Gavras H, Sultana R, Quackenbush J (2005). Independence and reproducibility across microarray platforms. Nat Methods.

[B22] Shi L, Tong W, Fang H, Scherf U, Han J, Puri RK, Frueh FW, Goodsaid FM, Guo L, Su Z, Han T, Fuscoe JC, Xu ZA, Patterson TA, Hong H, Xie Q, Perkins RG, Chen JJ, Casciano DA (2005). Cross-platform comparability of microarray technology: intra-platform consistency and appropriate data analysis procedures are essential. BMC Bioinformatics.

[B23] Hessner MJ, Meyer L, Tackes J, Muheisen S, Wang X (2004). Immobilized probe and glass surface chemistry as variables in microarray fabrication. BMC Genomics.

[B24] Hessner MJ, Wang X, Khan S, Meyer L, Schlicht M, Tackes J, Datta MW, Jacob HJ, Ghosh S (2003). Use of a three-color cDNA microarray platform to measure and control support-bound probe for improved data quality and reproducibility. Nucleic Acids Res.

[B25] Chou CC, Chen CH, Lee TT, Peck K (2004). Optimization of probe length and the number of probes per gene for optimal microarray analysis of gene expression. Nucleic Acids Res.

[B26] Relogio A, Schwager C, Richter A, Ansorge W, Valcarcel J (2002). Optimization of oligonucleotide-based DNA microarrays. Nucleic Acids Res.

[B27] Guo Z, Guilfoyle RA, Thiel AJ, Wang R, Smith LM (1994). Direct fluorescence analysis of genetic polymorphisms by hybridization with oligonucleotide arrays on glass supports. Nucleic Acids Res.

[B28] Ogle BM, Cascalho M, Joao C, Taylor W, West LJ, Platt JL (2003). Direct measurement of lymphocyte receptor diversity. Nucleic Acids Res.

[B29] Bonarius HP, Baas F, Remmerswaal EB, van Lier RA, Berge IJ, Tak PP, de Vries N (2006). Monitoring the T-cell receptor repertoire at single-clone resolution. PLoS ONE.

[B30] Diehl F, Grahlmann S, Beier M, Hoheisel JD (2001). Manufacturing DNA microarrays of high spot homogeneity and reduced background signal. Nucleic Acids Research.

[B31] Yassai M, Naumova E, Gorski J, Oksenberg JR (1997). Generation of TCR spectratypes by multiplex PCR for T cell repertoire analysis.

[B32] Wang X, Ghosh S, Guo SW (2001). Quantitative quality control in microarray image processing and data acquisition. Nucleic Acids Research.

[B33] Wang X, Jiang N, Feng X, Xie Y, Tonellato PJ, Ghosh S, Hessner MJ (2003). A novel approach for high-quality microarray processing using third-dye array visualization technology. IEEE Trans Nanobioscience.

